# Approximating net interactions among rigid domains

**DOI:** 10.1371/journal.pone.0195618

**Published:** 2018-04-09

**Authors:** Pouya Tavousi

**Affiliations:** 1 Mechanical Engineering Department, School of Engineering, University of Connecticut, Storrs, CT, United States of America; 2 Pharmaceutical Sciences Department, School of Pharmacy, University of Connecticut, Storrs, CT, United States of America; Universidade Nova de Lisboa Instituto de Tecnologia Quimica e Biologica, PORTUGAL

## Abstract

Many physical simulations aim at evaluating the net interaction between two rigid bodies, resulting from the cumulative effect of pairwise interactions between their constituents. This is manifested particularly in biomolecular applications such as hierarchical protein folding instances where the interaction between almost rigid domains directly influences the folding pathway, the interaction between macromolecules for drug design purposes, self-assembly of nanoparticles for drug design and drug delivery, and design of smart materials and bio-sensors. In general, the brute force approach requires quadratic (in terms of the number of particles) number of pairwise evaluation operations for any relative pose of the two bodies, unless simplifying assumptions lead to a collapse of the computational complexity. We propose to approximate the pairwise interaction function using a linear predictor function, in which the basis functions have separated forms, i.e. the variables that describe local geometries of the two rigid bodies and the ones that reflect the relative pose between them are split in each basis function. Doing so replaces the quadratic number of interaction evaluations for each relative pose with a one-time quadratic computation of a set of characteristic parameters at a preprocessing step, plus constant number of pose function evaluations at each pose, where this constant is determined by the required accuracy of approximation as well as the efficiency of the used approximation method. We will show that the standard deviation of the error for the net interaction is linearly (in terms of number of particles) proportional to the regression error, if the regression errors are from a normal distribution. Our results show that proper balance of the tradeoff between accuracy and speed-up yields an approximation which is computationally superior to other existing methods while maintaining reasonable precision.

## Introduction

At the very heart of every static or dynamic simulation, lies the evaluation of physical interactions such as different forms of energy and force. Predicting trajectories in a complex many-body system demands numerical solving of equations of motion which, in turn, involves evaluation of pairwise interactions between individual objects at different time steps. Oftentimes, a large portion of the processing time is dedicated to pairwise interactions, which takes quadratic time, presuming that each particle is influenced by every other particle present in the system. However, the nature of every particular problem enforces a set of restricting conditions which perhaps can be utilized to simplify the original problem. In the following, major categories of simplifying techniques are reviewed.

### Ignoring far interactions

**Fig 1:**
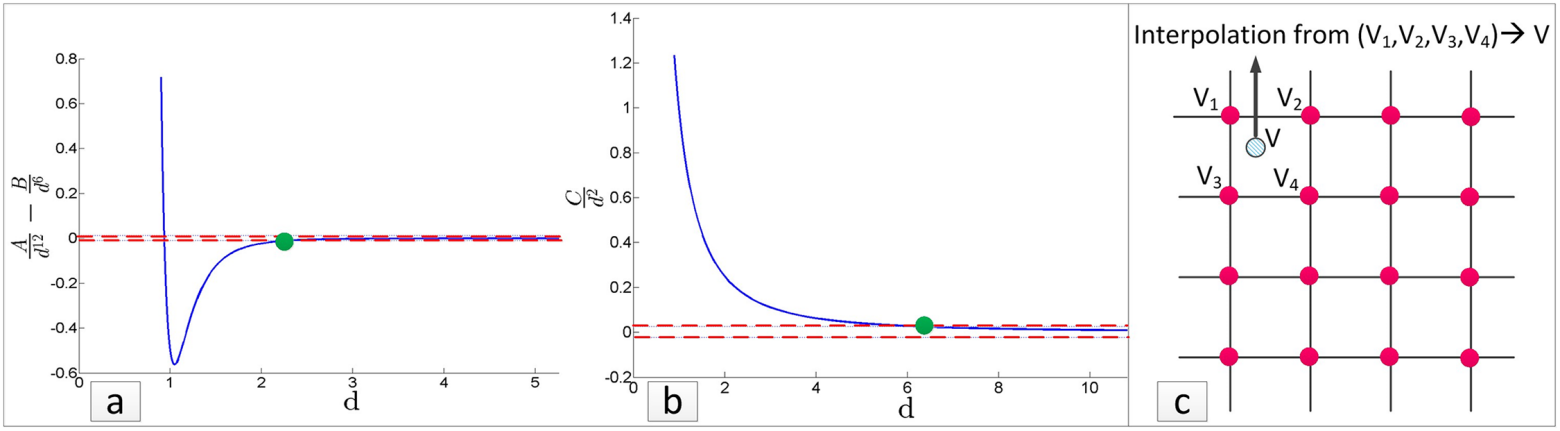
Existing techniques. Left: trend of pairwise interaction vs. pairwise distance for (a) van der Waals energy (b) electrostatic energy and/or gravity force (*A* = 1, *B* = 1.5 and *C* = 1). Within the dotted lines, pairwise interaction can be neglected and the green dot specifies the cut-off distance. Right: Interpolating from mesh values.

The profiles of several distance-dependent interactions suggest that the mutual effect of ‘far’ particles on each other can be neglected where, ‘far’ implies farther than a so-called cut-off distance, d which varies depending on the type of interaction. Fig 1a and 1b show this for different types of physical interactions. Assuming that electrostatic and van Der Waals force interactions between pairs of atoms fade away as their distance exceeds 9 and 5 Angstroms, respectively, [1] captures neighborhood information in an atomic ensemble using a *3D hash table*, leading to an *n*–fold reduction in force computation time.

### Using mesh values and interpolation

In many physical applications, the interaction profile does not experience abrupt changes anywhere throughout the domain under study. Particle-in-cell methods lay out a mesh on the computational domain to interpolate (Fig 1c) the values of pairwise interactions using the mesh values [2, 3]. Their computational complexity is *O*(*n* + *mlogn*), where *n* and *m* are the numbers of particles and mesh points, respectively [3]. In order to address the limited resolution provided by the mesh, the *P*^3^ *M* method introduced in [3] computes the short-range interactions directly, while for far-field interactions uses the mesh values. A similar approach is taken in [4], by replacing the mesh with a tree. By stating the potential at each point as a sum of three components, namely, far, near and external, the work in [2] uses multi-pole expansions to reduce the computation complexity down to *O*(*n*).

### Exploiting rigidity

Oftentimes, it is observed in physical systems that groups of objects are lumped together as rigid bodies. Examples of this phenomenon can be seen in hierarchic protein folding instances, where the interaction between almost rigid domains directly influences the folding pathway [5], the interaction between macromolecules [6–13] for drug design purposes, self-assembly of nanoparticles [14] for drug design and drug delivery applications, design of smart materials [15] and biosensors [16], as well as the interaction between stellar clusters [17]. In such cases, we look at the motion of each ensemble as a whole. Also, rather than evaluating single pairwise interactions, we are more interested in their net effect. The assumption of rigidity can be exploited in different ways:

#### Faster evaluation of kinematic parameters

Rigid body constraint algorithm is incorporated in [18] into a GPU-accelerated MD to speed up the numerical solving of equations of motion. Distance and angular holonomic constraints are enforced in [19] during the molecular simulations. Also, [20] suggests a robust parallelizable constraint method for molecular simulations. These methods, however, do not offer techniques for reducing the computational complexity associated with interaction computations.

#### Faster net interaction evaluation by avoiding pairwise evaluations within rigid bodies

In the brute force approach, computing the net interaction between a pair of rigid bodies is accomplished by simply adding up all the pairwise interactions. This can be however computationally improved when rigid domains are identified within the system. In an effort to computationally benefit from the rigidity condition, [1, 21–23] the protein molecule was modeled, in [1, 21–23] as a kinematic chain with groups of atoms lumped together. These groups of atoms act as rigid bodies or links and thus, lead to speedups in force computation and motion tracking in simulations, by neglecting the internal forces. However, since the number of rigid bodies is proportional to the number of atoms, the computational complexity remains dependent of the number of atoms. The *Chain Tree* data structure introduced in [24] stores the distance status of pairs in a hierarchy, allowing fast inquiry and update for them, avoiding interaction evaluations within rigid sections and resulting in a logarithmic reduction of time complexity (still dependent on the number of particles). Notice that, as long as the net effect of interactions on each individual particle is of concern, the minimum reachable computational complexity is of the order of *O*(*n*), n being the number of particles present in the system.

#### Faster net interaction evaluation by approximating attributes of rigid body pair

As long as the pairwise effect of interest is distance dependent, the net interaction can be expressed only as a function of the relative pose of the two bodies, plus a description of geometry at a reference relative pose. The number of independent variables needed for articulating the pose equals 6 in the most general case, with 3 translational and 3 rotational variables. However, this number can be as low as 1 if specific restraints are enforced such as the case studied in [14], where the two alpha helices are hinged together by means of a revolute joint. This suggests that unlike the general *n*− body problem (where the net effect of interactions on each particle must be tracked), a computational complexity that is independent of *n* is in theory achievable, due to the fact that number of parameters needed to describe the state of the systems collapses from *O*(*n*) to *O*(1) (= the number of relative pose parameters). Although for certain special cases with restrained geometries, closed-form simplifications of the interaction functions can be suggested, such as the case of gravity force between earth and a mass on its surface (where the earth is taken as a sphere and the mass on the surface is taken as a point), a straightforward formulation does not seem to exist for the general unrestrained geometry (i.e. between arbitrary shapes), as we may end up with expressions with a quadratic number of terms, each of which referring to a single pairwise interaction. In [25], a method is proposed for evaluation of long-range forces and moments between rigid bodies. For a prevalent form of the interaction function, where force is a negative integer power of distance, the pairwise interaction in [25] is approximated by obtaining the binomial series expansion of the force function followed by neglecting the terms that become insignificant when the two objects get “far enough” from each other. The method offers a computational complexity of interaction evaluation which is independent of the number of particles present in the simulation. However, observe that the method is limited to specific forms of force function (*F* ∝ 1/*d*^*n*^, where *d* is distance and *n* is a positive integer number). Therefore, other forms of force, for instance, the widely used linear and non-linear spring models [26, 27] for describing the interaction between particles do not fall within the scope of application of their method. In addition, the method only addresses the computation of long-range interactions and therefore fails to compute the net interaction when the two objects become closer than a threshold distance. Therefore, short-range interactions which play an important role in determining the overall attributes of the system must be treated separately with conventional approaches.

**Fig 2:**
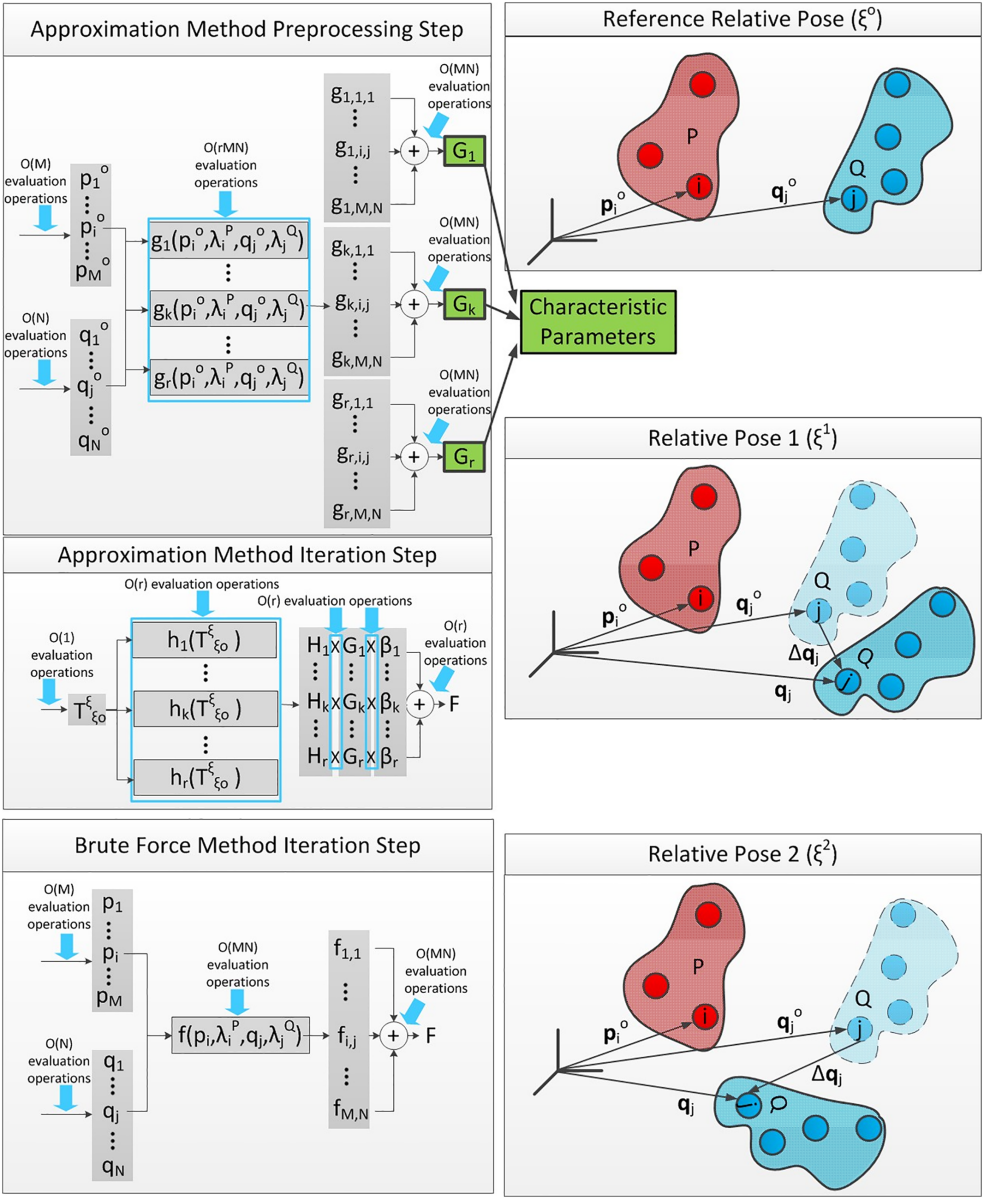
Brute force versus the proposed approximation method for computing the net interaction between two rigid bodies at multiple poses.

In this paper, we attempt to address the shortcomings of the existing methods in exploiting the rigidity condition. We propose to approximate the pairwise interaction function using a linear predictor function, in which the basis functions have separated forms [28], i.e. the variables that describe local geometries of the two rigid bodies and the ones that reflect the relative pose between them are split in each basis function. Doing so facilitates certain summation operations on the pairwise interactions during a preprocessing step, yielding fast evaluation of instantaneous net interaction whenever required. The multivariate pairwise interaction function is approximated by one that has the following *separated* form [28]: $f\approx \sum_{k=1}^{r}\beta_{k}g_{k}h_{k}$, where *h*_*k*_ only concerns the relative pose of the two rigid bodies, *g*_*k*_ is only descriptive of geometry at a reference relative pose, *r* is the separation rank (Fig 2) and *β*_*k*_ is the regression coefficient. The advantage of this is that, now, we can collect all the terms with similar *h*_*k*_ and sum over all the corresponding *β*_*k*_ *g*_*k*_ values (which can be obtained from the geometry at the reference relative pose) once, in a preprocessing step to attain a set of *characteristic parameters*. Then, computing the net interaction requires an evaluation of only *O*(*r*) terms, i.e. {*h*_*k*_|1 ≤ *k* ≤ *r*}, instead of one evaluation per each pair of particles. Using this method, the quadratic number of pairwise interaction evaluations (*O*(*rMN*), *M* and *N*, being the numbers of particles of the two bodies), in computing the net interaction at each relative pose, is replaced with one-time quadratic operations in preprocessing (*O*(*MN*)) plus constant pose function evaluations at each pose (*O*(*r*)), where *r* is independent of *M* and *N* and is only a function of the accuracy and efficiency of the used approximation method.

The two steps for arriving at a linear regression that approximates a multivariate pairwise interaction (for functions that admit this variable decomposition) are: (1) finding appropriate basis functions (i.e., {*h*_*k*_, *g*_*k*_|1 ≤ *k* ≤ *r*}); and (2) adjusting the regression coefficients (i.e., {*β*_*k*_|1 ≤ *k* ≤ *r*}). Note that the accuracy of pairwise interaction has direct influence on the accuracy of the resulting net interaction.

Several different approaches can be found in the literature for approximating multivariate functions. A review of finite sums decomposition methods in mathematical analysis can be found in [29]. In [30], decomposable functions of several variables are studied. The paper deals, in particular, with a function of three variables and claims that many of the results are extendable to more than three variables. The algorithm presented in [28] estimates a function of many variables from scattered data by approximating it as a sum of separable functions. The method is linear in the number of data points as well as the number of variables, which makes it suitable for large data sets in high dimensions. Also, [31] gives a method for decomposing smooth function *H* of *k* variables into a finite sum of products of *k* functions of single variables, as well as, conditions for the existence of special decompositions. Moreover, [32] reviews the recent advances in the use of separated representations.

Reviewing the existing approximate decomposition methods, two observations are made: (1) each one of these methods involves some sort of data-driven training and optimization. (2) each method best fits certain types of multivariate functions.

We propose an approximate decomposition method for the prevalent distance-dependent interactions i.e., for the cases that the pairwise interaction is stated only as a function of the pairwise distance and some constant (over all poses) scalar values (e.g., electrostatic, van der Waals, gravity, spring, collision, etc.) (Note that, the pairwise distance itself, is a multivariate function of up to 12 variables (where 6 variables describe the coordinates of a pair of points at the reference relative pose and another 6 describe the pose parameters)). Our method, first surrogates the interaction function as a polynomial of squared distance ($\sum_{k=0}^{n}a_{k}{\left(d^{2}\right)}^{k}$), using the linear regression method. This is followed by substituting *d*^2^ in the polynomial with its equivalent, in terms of reference geometry and pose variables, and finally expanding the polynomial to get a finite sum of products. It is worthwhile noting that, choosing *d*^2^ over *d* as the independent variable, is key to this method which, guarantees a finite number of terms, resulted from the expansion.

Using this method, the two aforementioned steps of approximation (i.e., choosing the basis functions and adjusting the regression coefficient) are conducted automatically, with minimal data-driven training. In fact, the only training that is performed here is the linear regression that is done to get the polynomial of *d*^2^ which, takes only fractions of a second, even for very large data. Moreover, the presented method is an essay-to-implement technique that manifests the promise of using separated representation for computing the net interaction between rigid bodies at different poses. The utility of the method will be shown using several test cases. Note that, the accuracy of the polynomial approximation highly depends on the degree of the polynomial and thus the complexity of representation. We plan to embed more efficient approximation techniques into the method in the future stages of this work.

The proposed usage of separable functions yields a fast approximation method for the net interaction between lumped masses whose complexity and accuracy are independent of the number of particles, but rather dependent on the efficiency of the used decomposition technique. The presented polynomial method, although not optimal, but offers a straightforward and almost generic (i.e., with no restriction on the form or the domain of the pairwise interaction function as long as the pairwise interaction admits polynomial regression) technique for decomposing distance-dependent interactions. Moreover, the presented approach facilitates the design process [33, 34] of desired behaviors in a system of rigid bodies, by giving the designer the option to manipulate the characteristic values. We demonstrate that, the standard deviation of the error for the net interaction is linearly (in terms of number of particles) proportional to the regression error, if the regression errors are from a normal distribution. In the Results section, we show that as the number of particles and the number of poses which interaction must be evaluated at go up, the computational superiority of the proposed method over the brute force (or other methods whose complexities are dependent on the number of particles) can be observed more vividly. The proposed method particularly finds application in molecular simulations where the interplay between molecular domains must be studied, as well as molecular design where molecular domain systems must be designed with certain desired behaviors.

## Materials and methods

In this section, we will first show how separated representation can be used for approximating the net interaction function. Then, the polynomial regression/expansion method for approximating distance dependent functions is discussed. The section is concluded with an error analysis of the proposed approximation method.

### Separated variable representation

Let $\left\{{\vec{p}}_{i}^{o}|i\leq M\right\}$ and $\left\{{\vec{q}}_{i}^{o}|i\leq N\right\}$ represent the coordinates of individual particles in rigid bodies *P* and *Q* respectively, at some reference relative pose, *ξ*^*o*^, of the two rigid bodies. Without the loss of generality, we assume body *P* to be fixed in the configuration space (i.e., the reference frame to be attached to body *P*), and therefore the up to 6 variables that describe the spatial configuration of *Q*, are also representative of the relative pose of the two bodies. More so, let $\left\{\lambda_{i}^{P}|i\leq M\right\}$ and $\left\{\lambda_{i}^{Q}|i\leq N\right\}$ reflect some pose-independent property of the individual particles that appears in the pairwise interaction function (e.g., particle charge). The interaction among particle *i* of *P* and particle *j* of *Q* at any relative pose *ξ* can be stated as:

$$\begin{array}{c} f_{i,j}=f\left({\vec{p}}_{i},\lambda_{i}^{P},{\vec{q}}_{j},\lambda_{j}^{Q}\right) \end{array}$$ (1)

or alternatively

$$\begin{array}{c} f_{i,j}=f\left({\vec{p}}_{i}^{o},\lambda_{i}^{P},{\vec{q}}_{j}^{o},\lambda_{j}^{Q},T_{\xi^{o}}^{\xi }\right) \end{array}$$ (2)

where $T_{\xi^{o}}^{\xi }$ is the transformation operator that takes *ξ*^*o*^ to *ξ*. Assume that the pairwise interaction can be approximated by sparable functions in the following form:

$$\begin{array}{c} f_{i,j}\approx \sum_{k=1}^{r}\beta_{k}g_{k}\left({\vec{p}}_{i}^{o},\lambda_{i}^{P},{\vec{q}}_{j}^{o},\lambda_{j}^{Q}\right)h_{k}\left(T_{\xi^{o}}^{\xi }\right) \end{array}$$ (3)

where *r* is the separation rank [28]. Then the net interaction between the two rigid bodies can be expressed as:

$$\begin{array}{c} F_{PQ}=\sum_{i=1}^{M}\sum_{j=1}^{N}f_{i,j}\approx \sum_{k=1}^{r}\left[\sum_{i=1}^{M}\sum_{j=1}^{N}\beta_{k}g_{k}\left({\vec{p}}_{i}^{o},\lambda_{i}^{P},{\vec{q}}_{j}^{o},\lambda_{j}^{Q}\right)\right]h_{k}\left(T_{\xi^{o}}^{\xi }\right) \end{array}$$ (4)

Defining interaction characteristic constant $C_{k}=\sum_{i=1}^{M}\sum_{j=1}^{N}\beta_{k}g_{k}\left({\vec{p}}_{i},\lambda_{i}^{P},{\vec{q}}_{j},\lambda_{j}^{Q}\right)$, we have

$$\begin{array}{c} F_{PQ}\approx \sum_{k=1}^{r}C_{k}h_{k}\left(T_{\xi^{o}}^{\xi }\right) \end{array}$$ (5)

Since interaction characteristic constants are independent of pose, they can be computed once and be inserted into Eq (5). Then for finding the net interaction at any arbitrary pose, *O*(*r*) different terms must be evaluated (Fig 2).

### Polynomial regression/expansion

It was shown in the previous section that, if one can get a separated representation of the pairwise interaction, they can use it towards formulating the net interaction between two lumped masses at different poses and defining the characteristic values. Now, we will see how this separated representation can be achieved by surrogating the interaction profile with a polynomial of squared distances and then expanding it. Using polynomial regression, we get:

$$\begin{array}{c} f\left(d,\lambda_{i}^{P},\lambda_{j}^{Q}\right)\approx s\left(\lambda_{i}^{P},\lambda_{j}^{Q}\right)\sum_{k=0}^{n}a_{k}{\left(d^{2}\right)}^{k} \end{array}$$ (6)

where *d* is the distance between ${\vec{p}}_{i}$ and ${\vec{q}}_{j}$, *s* is a function of $\lambda_{i}^{P}$ and $\lambda_{j}^{Q}$, *a*_*k*_ is polynomial regression coefficient and *n* is the degrees of the polynomial. In the case that the left hand side of Eq (6) represents the magnitude of a vector function (i.e., $|\vec{f}\left(d,\lambda_{i}^{P},\lambda_{j}^{Q}\right)|$) which is applied in the direction of the vector connecting ${\vec{p}}_{i}$ and ${\vec{q}}_{j}$ (e.g, pairwise force), the approximation can take place componentwise, giving rise to a proper vector sum for evaluating the net interaction. For that, instead of the interaction magnitude, the ratio of interaction magnitude over distance, is surrogated as a polynomial of squared distance:

$$\begin{array}{c} \frac{|\vec{f}\left(d,\lambda_{i}^{P},\lambda_{j}^{Q}\right)|}{d}\approx s\left(\lambda_{i}^{P},\lambda_{j}^{Q}\right)\sum_{k=0}^{n}a_{k}{\left(d^{2}\right)}^{k}, \end{array}$$ (7)

from which, components of the pairwise interaction can be attained:

$$\begin{array}{c} f_{i,j,l}=\frac{|\vec{f}\left(d,\lambda_{i}^{P},\lambda_{j}^{Q}\right)|}{d}\left(q_{j,l}-p_{i,l}^{o}\right) \end{array}$$ (8)

where,

$$\begin{array}{c} {\vec{q}}_{j}=T_{\xi^{o}}^{\xi }{\vec{q}}_{j}^{o} \end{array}$$ (9)

and subscript *l* takes values 1, 2 or 3 which, respectively, indicate *x*, *y* and *z* components. Also, in this method, certain additional treatments of the pairwise interaction, before conducting the summation over pairs, are allowed as long as the polynomial form is preserved. For instance, one can approximate the pairwise moment by manipulating the pairwise force interaction:

$$\begin{array}{c} {\vec{m}}_{i,j}={\vec{q}}_{j}\times {\vec{f}}_{i,j}=\left(T_{\xi^{o}}^{\xi }{\vec{q}}_{j}^{o}\right)\times {\vec{f}}_{i,j} \end{array}$$ (10)

The right hand sides of Eq (6) can expanded after the following replacement:

$$\begin{array}{c} d^{2}=\left({\vec{q}}_{j}-{\vec{p}}_{i}^{o}\right)\cdot \left({\vec{q}}_{j}-{\vec{p}}_{i}^{o}\right)=\sum_{l=1}^{3}{\left(q_{j,l}-p_{i,l}^{o}\right)}^{2} \end{array}$$ (11)

and using Eq (9) to substitute ${\vec{q}}_{j}$. In the most general case (where all 6 pose parameters are variable), we can express the transformation as a combination of rotation by an angle *θ* about an axis in the direction of a unit vector $\vec{u}={\left(u_{1}u_{2}u_{3}\right)}^{t}$ where $u_{1}^{2}+u_{2}^{2}+u_{3}^{2}=1$ and a translation vector $\vec{x}={\left(x_{1}x_{2}x_{3}\right)}^{t}$ (superscript ^t^ indicates transpose.):

$$\begin{array}{c} T_{\xi_{0}}^{\xi }=\left[\begin{array}{cccc} cos\theta +u_{1}^{2}\left(1-cos\theta \right) & u_{1}u_{2}\left(1-cos\theta \right)-u_{3}sin\theta & u_{1}u_{3}\left(1-cos\theta \right)+u_{2}sin\theta & x_{1}\\ u_{2}u_{1}\left(1-cos\theta \right)+u_{3}sin\theta & cos\theta +u_{2}^{2}\left(1-cos\theta \right) & u_{2}u_{3}\left(1-cos\theta \right)-u_{1}sin\theta & x_{2}\\ u_{3}u_{1}\left(1-cos\theta \right)-u_{2}sin\theta & u_{3}u_{2}\left(1-cos\theta \right)+u_{1}sin\theta & cos\theta +u_{3}^{2}\left(1-cos\theta \right) & x_{3}\\ 0 & 0 & 0 & 1 \end{array}\right] \end{array}$$

Substituting this into Eq (9), we have

$$\begin{array}{c} {\vec{q}}_{j}=\left(\begin{array}{c} \left(cos\theta +u_{1}^{2}\left(1-cos\theta \right)\right)q_{j,1}^{o}+\left(u_{1}u_{2}\left(1-cos\theta \right)-u_{3}sin\theta \right)q_{j,2}^{o}+\left(u_{1}u_{3}\left(1-cos\theta \right)+u_{2}sin\theta \right)q_{j,3}^{o}+x_{1}\\ \left(u_{2}u_{1}\left(1-cos\theta \right)-u_{3}sin\theta \right)q_{j,1}^{o}+\left(cos\theta +u_{2}^{2}\left(1-cos\theta \right)\right)q_{j,2}^{o}+\left(u_{2}u_{3}\left(1-cos\theta \right)-u_{1}sin\theta \right)q_{j,3}^{o}+x_{2}\\ \left(u_{3}u_{1}\left(1-cos\theta \right)-u_{2}sin\theta \right)q_{j,1}^{o}+\left(u_{3}u_{2}\left(1-cos\theta \right)+u_{1}sin\theta \right)q_{j,1}^{o}+\left(cos\theta +u_{3}^{2}\left(1-cos\theta \right)\right)q_{j,1}^{o}+x_{3} \end{array}\right) \end{array}$$

Consequently, substituting this in (11) gives:

$$\begin{array}{ccc} d^{2} & = & {\left(\begin{array}{c} q_{j,1}^{o} \end{array}\begin{array}{c} \left(cos\theta +u_{1}^{2}\left(1-cos\theta \right)\right) \end{array}+\begin{array}{c} q_{j,2}^{o} \end{array}\begin{array}{c} \left(u_{1}u_{2}\left(1-cos\theta \right)-u_{3}sin\theta \right) \end{array}+\begin{array}{c} q_{j,3}^{o} \end{array}\begin{array}{c} \left(u_{1}u_{3}\left(1-cos\theta \right)+u_{2}sin\theta \right) \end{array}+\begin{array}{c} x_{1} \end{array}\right)}^{2}\\ & + & {\left(\begin{array}{c} q_{j,1}^{o} \end{array}\begin{array}{c} \left(u_{2}u_{1}\left(1-cos\theta \right)+u_{3}sin\theta \right) \end{array}+\begin{array}{c} q_{j,2}^{o} \end{array}\begin{array}{c} \left(cos\theta +u_{2}^{2}\left(1-cos\theta \right)\right) \end{array}+\begin{array}{c} q_{j,3}^{o} \end{array}\begin{array}{c} \left(u_{2}u_{3}\left(1-cos\theta \right)-u_{1}sin\theta \right) \end{array}+\begin{array}{c} x_{2} \end{array}\right)}^{2}\\ & + & {\left(\begin{array}{c} q_{j,1}^{o} \end{array}\begin{array}{c} \left(u_{3}u_{1}\left(1-cos\theta \right)-u_{2}sin\theta \right) \end{array}+\begin{array}{c} q_{j,1}^{o} \end{array}\begin{array}{c} \left(u_{3}u_{2}\left(1-cos\theta \right)+u_{1}sin\theta \right) \end{array}+\begin{array}{c} q_{j,1}^{o} \end{array}\begin{array}{c} \left(cos\theta +u_{3}^{2}\left(1-cos\theta \right)\right) \end{array}+\begin{array}{c} x_{3} \end{array}\right)}^{2} \end{array}$$

Note that *d*^2^ is now represented as a sum of squares of separated representations. We will now show that this can turn into a separated representation.

**Lemma 1.** Let function *f* have a separated representation. Then, *f*^*n*^, in which *n* is a non-negative integer, also has a separated representation.

**Proof.** Suppose that *f* has the following form:

$$\begin{array}{c} f=\sum_{k=1}^{r}g_{k}h_{k} \end{array}$$ (12)

Then

$$\begin{array}{c} f^{n}={\left(\sum_{k=1}^{r}g_{k}h_{k}\right)}^{n}={\left(g_{1}h_{1}+g_{2}h_{2}+...+g_{k}h_{k}+...+g_{r}h_{r}\right)}^{n} \end{array}$$ (13)

$$\begin{array}{c} f^{n}=\sum_{\forall (\alpha_{1}+...+\alpha_{r}=n)}C_{\alpha_{1},...,\alpha_{r}}\prod_{k=1}^{r}{g_{k}}^{\alpha_{k}}{h_{k}}^{\alpha_{k}}=\sum_{\forall (\alpha_{1}+...+\alpha_{r}=n)}C_{\alpha_{1},...,\alpha_{r}}\begin{array}{c} {g_{1}}^{\alpha_{1}}{g_{2}}^{\alpha_{2}}...{g_{k}}^{\alpha_{k}}...{g_{r}}^{\alpha_{r}} \end{array}\begin{array}{c} {h_{1}}^{\alpha_{1}}{h_{2}}^{\alpha_{2}}...{h_{k}}^{\alpha_{k}}...{h_{r}}^{\alpha_{r}} \end{array} \end{array}$$ (14)

where *α*_1_, …, *α*_*r*_ are non-negative integer numbers and

**Fig 3:**
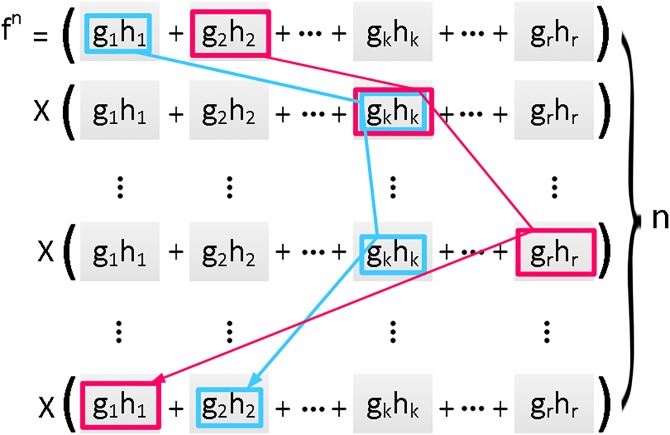
Each path results in an algebraic term that has a separated form.

Cα1,...,αr=(nα1)(n-α1α2)...(n-(α1+α2+...+αr-1)αr)

where (nk) indicates the number of *k*-combinations from a set of *n* elements (Fig 3).

From, it can be concluded that, *d*^2^ and consequently, $\sum_{k=0}^{n}a_{k}{\left(d^{2}\right)}^{k}$ have separated forms.

Here as an example, we derive the separated formulation for *d*^2^ for the case that, only two out of six pose parameters, *x*_1_ and *θ* are variable (i.e., *x*_2_ = *x*_3_ = *u*_1_ = *u*_2_ = 0, *u*_3_ = 1). The transformation will take the following form:

$$\begin{array}{c} T_{\xi_{0}}^{\xi }=\left[\begin{array}{cccc} cos\theta & -sin\theta & 0 & x_{1}\\ sin\theta & cos\theta & 0 & 0\\ 0 & 0 & 1 & 0\\ 0 & 0 & 0 & 1 \end{array}\right] \end{array}$$

Replacing this in 9 yields:

$$\begin{array}{c} {\vec{q}}_{j}=\left(\begin{array}{c} cos\theta q_{j,1}^{o}-sin\theta q_{j,2}^{o}+x_{1}\\ sin\theta q_{j,1}^{o}+cos\theta q_{j,2}^{o}\\ q_{j,3}^{o} \end{array}\right) \end{array}$$

Substituting this in 11 gives:

$$\begin{array}{c} d^{2}={\left(cos\theta q_{j,1}^{o}-sin\theta q_{j,2}^{o}+x_{1}-p_{i,1}^{o}\right)}^{2}+{\left(sin\theta q_{j,1}^{o}+cos\theta q_{j,2}^{o}-p_{i,2}^{o}\right)}^{2}+{\left(q_{j,3}^{o}-p_{i,3}^{o}\right)}^{2} \end{array}$$ (15)

or

$$\begin{array}{ccc} d^{2} & = & \begin{array}{c} x_{1}^{2} \end{array}+\begin{array}{c} {p_{i,1}^{o}}^{2}+{p_{i,2}^{o}}^{2}+{\left(q_{j,3}^{o}-p_{i,3}^{o}\right)}^{2} \end{array}+\begin{array}{c} -p_{i,1}^{o} \end{array}\begin{array}{c} 2x_{1} \end{array}+\begin{array}{c} {q_{j,1}^{o}}^{2}+{q_{j,2}^{o}}^{2} \end{array}\begin{array}{c} cos^{2}\theta \end{array}+\begin{array}{c} {q_{j,1}^{o}}^{2}+{q_{j,2}^{o}}^{2} \end{array}\begin{array}{c} sin^{2}\theta \end{array}\\ & + & \begin{array}{c} -q_{j,1}^{o}p_{i,1}^{o}-q_{j,2}^{o}p_{i,2}^{o} \end{array}\begin{array}{c} 2cos\theta \end{array}+\begin{array}{c} q_{j,2}^{o}p_{i,1}^{o}-q_{j,1}^{o}p_{i,2}^{o} \end{array}\begin{array}{c} 2sin\theta \end{array}+\begin{array}{c} q_{j,1}^{o} \end{array}\begin{array}{c} 2cos\theta x_{1} \end{array}+\begin{array}{c} -q_{j,2}^{o} \end{array}\begin{array}{c} 2sin\theta x_{1} \end{array} \end{array}$$

which as can be observed, has a separated form.

### Error analysis

Every approximation introduces some type of error. We must see how the error of pairwise interaction contributes to the error of the net approximation. Eq (3) can be restated as

$$\begin{array}{c} f_{i,j}=\sum_{k=1}^{r}\beta_{k}g_{k}\left({\vec{p}}_{i}^{o},\lambda_{i}^{P},{\vec{q}}_{j}^{o},\lambda_{j}^{Q}\right)h_{k}\left(T_{\xi^{o}}^{\xi }\right)+\epsilon_{i,j} \end{array}$$ (16)

where, *ϵ*_*i*,*j*_ is the regression error. This reformulates Eq (4) in the following form:

$$\begin{array}{c} F_{PQ}=\sum_{k=1}^{r}\left[\sum_{i=1}^{M}\sum_{j=1}^{N}\beta_{k}g_{k}\left({\vec{p}}_{i}^{o},\lambda_{i}^{P},{\vec{q}}_{j}^{o},\lambda_{j}^{Q}\right)\right]h_{k}\left(T_{\xi^{o}}^{\xi }\right)+E_{net} \end{array}$$ (17)

where $E_{net}=\sum_{i=1}^{M}\sum_{j=1}^{N}\epsilon_{i,j}$. Assuming a normal distribution for the regression error ($\epsilon_{i,j}∼N\left(0,\sigma_{r}^{2}\right)$), we have

$$\begin{array}{c} E∼N\left(0,MN\sigma_{r}^{2}\right)=N\left(0,{\left(\sqrt{MN}\sigma_{r}\right)}^{2}\right) \end{array}$$ (18)

implying that the error for the net interaction is linearly (in terms of number of particles) proportional to the regression error, if the regression errors are from a normal distribution.

## Results and discussion

This section provides a few examples in which, the polynomial regression/expansion method is used to obtain a separated representation (with only one variable pose parameter) of a few types of pairwise interactions, followed by using it to evaluate the net interaction between lumped masses at different poses. We will observe that the computational time collapses from quadratic to constant yet, the resulting accuracy is reasonable.

### Electrostatic energy of two rectangular cubes, 1D translation

**Fig 4:**
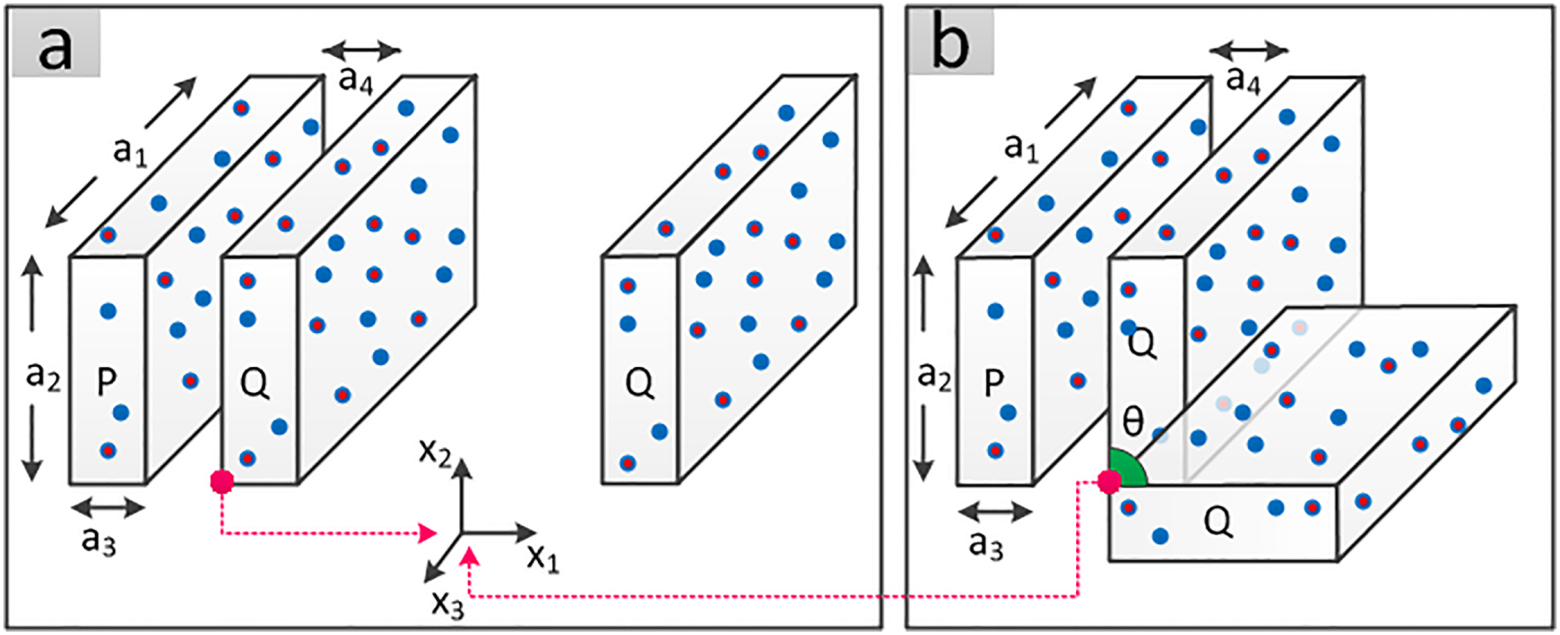
Object P is fixed. Two scenarios occur for object Q: (a) it is translated in *x*_1_ direction; (b) it is rotated about *x*_3_.

Consider the two rectangular cubes *P* and *Q* shown in Fig 4a, where point charges are distributed inside the objects. Knowing the local coordinates of each point charge inside each object, we want to evaluate the electrostatic energy of the system at a sequence of snapshots as *Q* travels a certain distance in *x*_1_ direction. The pairwise electrostatic energy for two point charges $\lambda_{i}^{P}$ and $\lambda_{j}^{Q}$ is

$$\begin{array}{c} e_{i,j}=\frac{\lambda_{i}^{P}\lambda_{j}^{Q}}{4\pi \epsilon d} \end{array}$$ (19)

where *d* is the distance between the charges and *ϵ* is the dielectric constant. Given that the only variable pose parameter here is *x*_1_, we have:

$$\begin{array}{c} d^{2}=\begin{array}{c} x_{1}^{2} \end{array}+\begin{array}{c} {\left(q_{j,1}^{o}-p_{i,1}^{o}\right)}^{2}+{p_{i,2}^{o}}^{2}+{p_{i,3}^{o}}^{2} \end{array}+\begin{array}{c} \left(q_{j,1}^{o}-p_{i,1}^{o}\right) \end{array}\begin{array}{c} 2x_{1} \end{array} \end{array}$$ (20)

**Fig 5:**
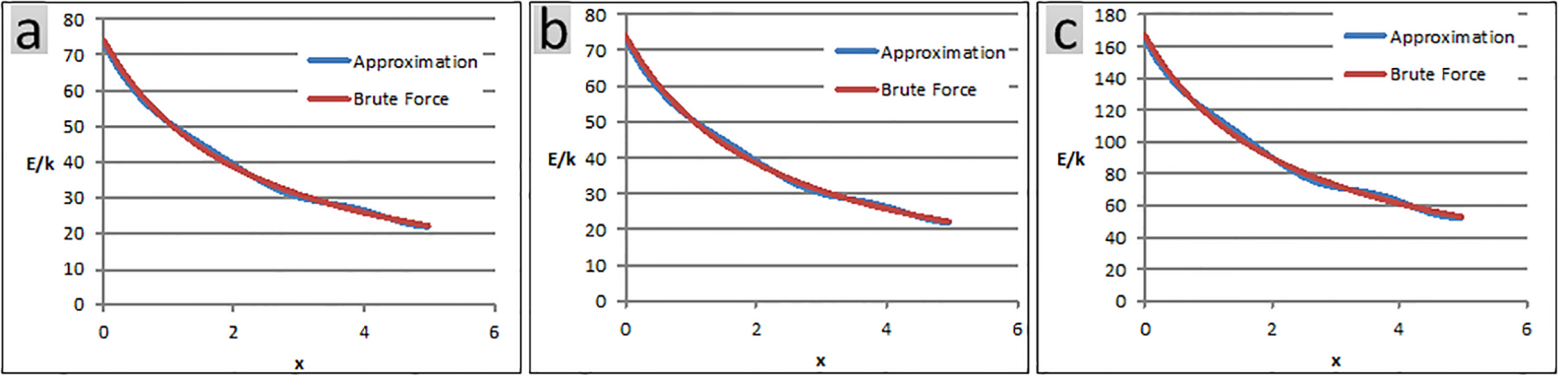
(a) Each object has 500 charged particles, randomly distributed within its confining box. The net electrostatic energy is evaluated at 500 snapshots. Conventional brute force method takes 40s of processor time compared to 6s taken by the approximation method. The average error is 1.75%. (b) Objects are similar to case a. Energy is evaluated in 100 snapshots. Conventional brute force method takes 8s long, while the approximation method is accomplished in 6s. The average error is 1.75%. (c) Each object has 1000 charged particles, randomly distributed within its confining box. Energy evaluation is conducted at 500 snapshots. Conventional brute force and approximation methods last 160s and 24s respectively. The average error is 2.15%.

We need to approximate the function 1/*d* as a polynomial of *d*^2^. Let *a*_1_ = *a*_2_ = 3 and *a*_3_ = *a*_4_ = 1 for this example. Also, let 0 ≤ *x*_1_ ≤ 5. It can be shown that for this special case, 1 ≤ *d*^2^ ≤ 82 and thus the regression needs to be valid for this range. Choosing *n* = 9 as the degree of the polynomial regression, substituting 20 in 6 and conducting polynomial expansion results in the following expression for the pairwise energy:

$$\begin{array}{c} e_{i,j}=\sum_{k=0}^{18}\beta_{k,i,j}x_{1}^{k} \end{array}$$ (21)

where, *β*_*k*,*i*,*j*_ is only a function of ${\vec{p}}_{i}^{o}$, $\lambda_{i}^{P}$, ${\vec{q}}_{j}^{o}$ and $\lambda_{j}^{Q}$. Fig 5 juxtaposes the values obtained for the net energy obtained from the conventional brute force method and the proposed approximation technique, as well as the time spent by each method for 3 different test cases.

**Fig 6:**
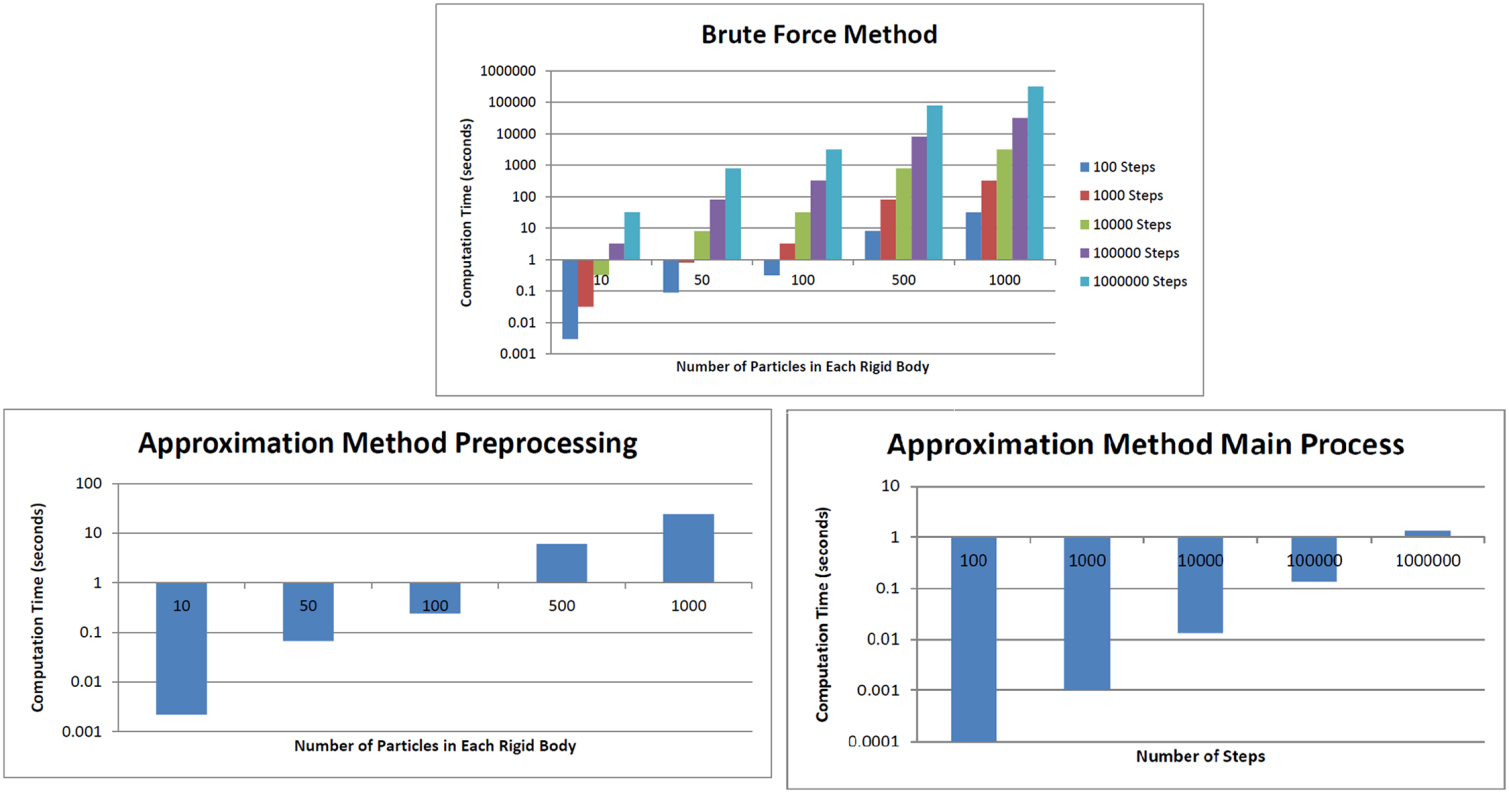
Computational time of conventional brute force method and approximation method for different selections of the number of steps and the number of particles in each rigid body.

Fig 6 compares the computational time of the conventional brute force method and the approximation method (preprocessing step as well as iterations for different relative poses) for different selections of two parameters, namely the number of particles in each rigid body and number of steps at which net interaction (energy in this case) is evaluated. For the proposed method, the overall computation time is obtained by summing up the preprocessing time and that of the main process (i.e., iterations for different relative poses). Note that the number of particles only affect the former and the number of iterations (i.e., the number of poses at which the net interaction must be evaluated) only affects the latter.

### Electrostatic energy of two rectangular cubes, 1*D* rotation

Now, let object *Q* be rotated about *x*_3_ axis. This time, given that the only variable pose parameter is *θ*, we have:

$$\begin{array}{c} \begin{array}{ccc} d^{2} & = & \begin{array}{c} {p_{i,1}^{o}}^{2}+{p_{i,2}^{o}}^{2}+{\left(q_{j,3}^{o}-p_{i,3}^{o}\right)}^{2} \end{array}+\begin{array}{c} {q_{j,1}^{o}}^{2}+{q_{j,2}^{o}}^{2} \end{array}\begin{array}{c} cos^{2}\theta \end{array}+\begin{array}{c} {q_{j,1}^{o}}^{2}+{q_{j,2}^{o}}^{2} \end{array}\begin{array}{c} sin^{2}\theta \end{array}\\ & + & \begin{array}{c} -q_{j,1}^{o}p_{i,1}^{o}-q_{j,2}^{o}p_{i,2}^{o} \end{array}\begin{array}{c} 2cos\theta \end{array}+\begin{array}{c} q_{j,2}^{o}p_{i,1}^{o}-q_{j,1}^{o}p_{i,2}^{o} \end{array}\begin{array}{c} 2sin\theta \end{array} \end{array} \end{array}$$

**Fig 7:**
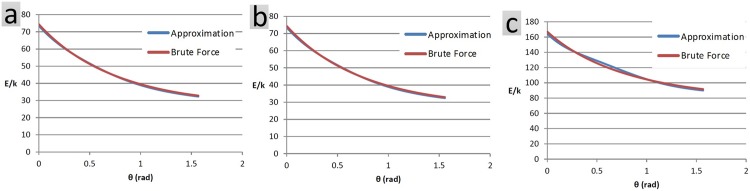
(a) Each object has 500 charged particles, randomly distributed within its confining box (same objects as the case of translation example). The net electrostatic energy is evaluated at 500 snapshots. Conventional brute force method takes 40s of processor time compared to 21s taken by the approximation method. The average error is 0.99%. (b) Objects are similar to case a. Energy is evaluated in 100 snapshots. Conventional brute force method takes 8s long, while the approximation method is accomplished in 21s. The average error is 0.99%. (c) Each object has 1000 charged particles, randomly distributed within its confining box. Energy evaluation is conducted at 500 snapshots. Conventional brute force and approximation methods last 159s and 84s respectively. The average error is 1.57%.

Let the box dimensions be the same as the previous case. Also, let −*π*/2 ≤ *θ* ≤ 0. Again, choosing *n* = 9 as the degree of the polynomial regression, substituting the expression for *d*^2^ in 6, conducting polynomial expansion and noting that, *cos*^2^ *θ* = 1 − *sin*^2^ *θ*, results in the following expression for the pairwise energy:

$$\begin{array}{c} e_{i,j}=\sum_{k=0}^{9}\beta_{k,i,j}{\left(sin\theta \right)}^{k}+\gamma_{k,i,j}{\left(sin\theta \right)}^{k}cos\theta \end{array}$$ (22)

where, *β*_*k*,*i*,*j*_ and *γ*_*k*,*i*,*j*_ are only functions of ${\vec{p}}_{i}^{o}$, $\lambda_{i}^{P}$, ${\vec{q}}_{j}^{o}$ and $\lambda_{j}^{Q}$. Similar to the previous case, Fig 7 compares the performances of conventional brute force and approximation method.

### Almost-rigid bodies

**Fig 8:**
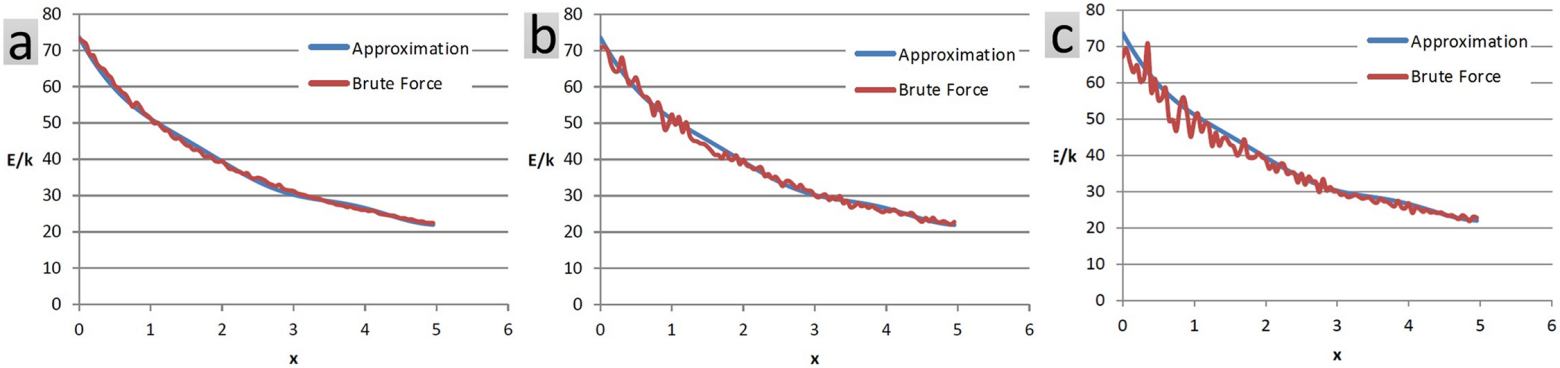
The translation example is repeated, but this time, we let particles deviate from their initial local coordinates at the reference pose, during the motion. The deviation is confined by a cube whose edge takes a certain size and is 0.1 for case a, 0.3 for case b and 0.5 for case c. The average errors for the three cases are respectively 1.77%, 2.58% and 4.05%.

In several real cases, the objects under study do not remain absolutely rigid during their range of motion but rather fluctuate around a so-called average rigid structure. Such phenomenon is often seen in molecular domains and usually is reflected by an index called B factor [35]. To study the effect of flexibility factor on the performance of the approximation method, we repeat the first example for 3 different levels of rigidity. The brute force method computes the net interaction based on the exact coordinates of the particles at each snapshot, while the approximation method assumes a rigid body motion (Fig 8).

### Net force and net moment approximation

**Fig 9:**
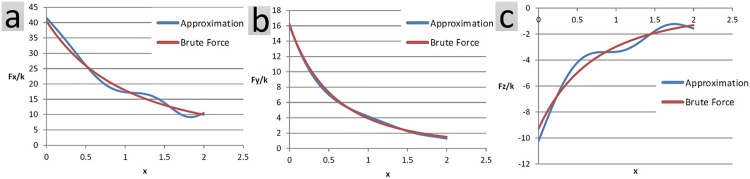
Components of the net force are computed using the approximation technique as well as the conventional brute force method. Each object has 500 charged particles, randomly distributed within its confining box. Net force evaluation is conducted at 500 snapshots. The conventional method takes 55s of processor time compared to 8s taken by the approximation method. The average error is 6.99%.

**Fig 10:**
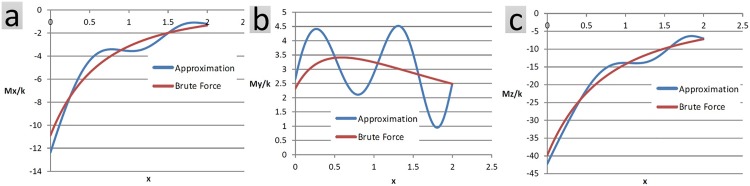
Components of the net moment are computed using the approximation technique as well as the conventional brute force method. Each object has 500 charged particles, randomly distributed within its confining box. The net moment evaluation is conducted at 500 snapshots. The conventional method takes 55s of processor time compared to 8s taken by the approximation method. The average error is 12.37%.

Approximations of net force and net moment are shown using another example. Let *a*_1_ = *a*_2_ = *a*_3_ = *a*_4_ = 1 reflect the dimensions and 0 ≤ *x*_1_ ≤ 2 for the objects of Fig 4a. Figs 9 and 10 compare the approximation technique with the brute force method in computing different components of the net force as well as the net moment between the two objects at different snapshots.

### Electrostatic energy of two alpha helices, 1-D translation

**Fig 11:**
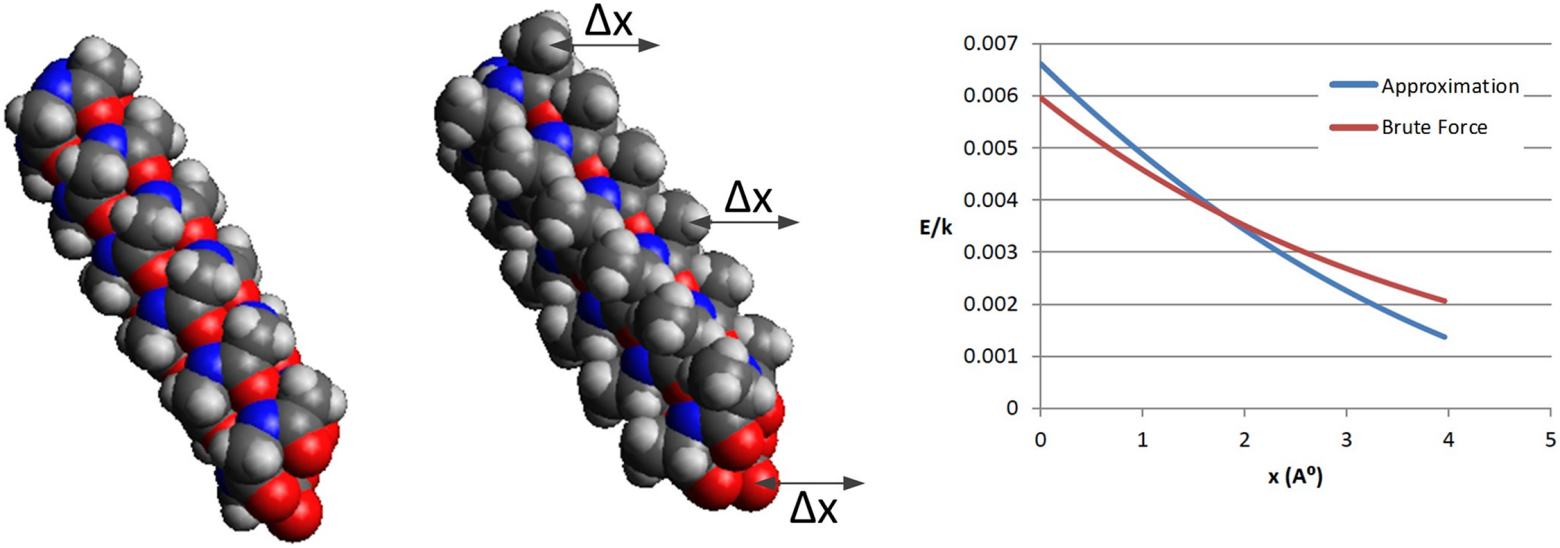
Left is an all-glycine alpha helix with 25 residues and is fixated in space. Right is an all-Alanine alpha helix again with 25 residues and is translated 4*Å* in the x direction. The conventional and brute force evaluations of the electrostatic energy between the two object are shown. The average error is 11.26%.

Fig 11 compares the two methods in evaluating the net interaction between two alpha helices. The first helix is fixated in the space, while the other one is translated 4*Å* in the x direction. The electrostatic energy resulting from the partial charges on the atoms of the two objects is computed at different snapshots during the motion, both using the conventional brute force method and the suggested approximation technique.

## Conclusion

Despite all the breakthroughs in the field of computer algorithms, as well as the huge computational power available on even personal computers, the quadratic computational complexity associated with exact pairwise interaction evaluations remains the bottleneck of static and dynamic simulations. This has caused an ongoing search for efficient approximation methods. In this paper, we proposed an approximation method for the special case that the net interaction between two rigid bodies, resulting from the cumulative effect of pairwise interactions between their constituents, must be evaluated. A linear predictor, the basis functions of which have separated forms, is used to approximate the values of a multivariate interaction function. In other words, the variables that describe the local geometries of the two rigid bodies and the ones that reflect the relative pose between them are split. This facilitates certain summation operations, when computing the net interaction. As a result, the quadratic number of interaction evaluations for each relative pose is replaced with a one-time quadratic computation of a set of characteristic parameters at a preprocessing step, plus constant number of pose function evaluations at each pose, where this constant is determined by the required accuracy of approximation as well as the efficiency of the used approximation method. We showed that the standard deviation of the error for the net interaction is linearly proportional to the regression error, if the regression errors are from a normal distribution. Our results also showed that even exploiting the simple polynomial regression/expansion method to obtain the separated representation can yield faster computation yet comparable in accuracy to the conventional brute force method. This promises even better performances if more efficient fitting algorithms are employed.
